# Beyond the Exome: The Non-coding Genome and Enhancers in Neurodevelopmental Disorders and Malformations of Cortical Development

**DOI:** 10.3389/fncel.2019.00352

**Published:** 2019-07-31

**Authors:** Elena Perenthaler, Soheil Yousefi, Eva Niggl, Tahsin Stefan Barakat

**Affiliations:** Department of Clinical Genetics, Erasmus MC – University Medical Center, Rotterdam, Netherlands

**Keywords:** malformations of cortical development, gene regulation, cis-regulatory elements, enhancers, epigenome, functional genomics, massively parallel reporter assays, bioinformatics

## Abstract

The development of the human cerebral cortex is a complex and dynamic process, in which neural stem cell proliferation, neuronal migration, and post-migratory neuronal organization need to occur in a well-organized fashion. Alterations at any of these crucial stages can result in malformations of cortical development (MCDs), a group of genetically heterogeneous neurodevelopmental disorders that present with developmental delay, intellectual disability and epilepsy. Recent progress in genetic technologies, such as next generation sequencing, most often focusing on all protein-coding exons (e.g., whole exome sequencing), allowed the discovery of more than a 100 genes associated with various types of MCDs. Although this has considerably increased the diagnostic yield, most MCD cases remain unexplained. As Whole Exome Sequencing investigates only a minor part of the human genome (1–2%), it is likely that patients, in which no disease-causing mutation has been identified, could harbor mutations in genomic regions beyond the exome. Even though functional annotation of non-coding regions is still lagging behind that of protein-coding genes, tremendous progress has been made in the field of gene regulation. One group of non-coding regulatory regions are enhancers, which can be distantly located upstream or downstream of genes and which can mediate temporal and tissue-specific transcriptional control via long-distance interactions with promoter regions. Although some examples exist in literature that link alterations of enhancers to genetic disorders, a widespread appreciation of the putative roles of these sequences in MCDs is still lacking. Here, we summarize the current state of knowledge on *cis*-regulatory regions and discuss novel technologies such as massively-parallel reporter assay systems, CRISPR-Cas9-based screens and computational approaches that help to further elucidate the emerging role of the non-coding genome in disease. Moreover, we discuss existing literature on mutations or copy number alterations of regulatory regions involved in brain development. We foresee that the future implementation of the knowledge obtained through ongoing gene regulation studies will benefit patients and will provide an explanation to part of the missing heritability of MCDs and other genetic disorders.

## Introduction

The brain lies at the foundation of what makes us human, as it not only regulates most of our body functions, but it is also central to our cognition and thoughts, defining our personalities, behavior and social interactions. How such a complex organ is formed during development has fascinated biologists for centuries, and we are now living in a technology driven era where knowledge gained through various disciplines such as medicine, biotechnology, computational biology, and neuroscience enables us for the first time to get a glimpse on how these intricate processes are genetically regulated. Understanding the developmental biology of the human brain is not only of utmost importance for satisfying our own natural curiosity of what makes us human, but also promises improvements and therapeutic options for various disorders that affect the human brain, including neurodevelopmental and neurodegenerative diseases, which, together, are a burden on society and have a negative effect on the quality of life of individuals (Genereaux et al., 2015; Jonsson et al., 2017). One of the traditionally best studied parts of the human brain is the cerebral cortex, which is responsible for cognition and sensorimotor activity. The development of the cerebral cortex is a complex and dynamic process organized in three major steps: (I) neural stem cell proliferation, (II) neuronal migration toward the cortical plate, and (III) post-migratory organization (for further review see, Agirman et al., 2017; Van Essen et al., 2018; Seto and Eiraku, 2019). Alterations in any of these steps can be responsible for the development of a group of disorders overall known as malformations of cortical development (MCD), a common cause of neurodevelopmental delay, intellectual disability and epilepsy (Guerrini and Dobyns, 2014).

Malformations of cortical development are a heterogeneous group of disorders originally classified into three main groups, reflecting the stage of cortical development at which the defect arises (Barkovich et al., 1996). To date, more than a 100 genes associated with MCDs have been described (Guerrini and Dobyns, 2014), the majority of which encodes for proteins that function in general neurodevelopmental processes such as cell cycle regulation, neuronal migration, and polarization. These include classically studied MCD genes such as *LIS1*, that, when mutated, causes lissencephaly (Reiner et al., 1993), but also a flood of recently identified genes such as *TLE1* (Cavallin et al., 2018), *GRIN1* (Fry et al., 2018), *CRADD* (Di Donato et al., 2016), *DIAPH1* (Ercan-Sencicek et al., 2015), *WDR62* (Bilguvar et al., 2010), *RTTN* (Kheradmand Kia et al., 2012), *ZIC1* (Vandervore et al., 2018), *MACF1* (Dobyns et al., 2018), and many more (Lu et al., 2018) that could only be implicated in MCD due to the advent of next-generation DNA sequencing technologies, which enable large-scale genomic investigations in an unbiased, hypothesis-free manner.

Potential disease-causing genetic mutations can be detected by whole exome sequencing (WES), a method of sequencing all protein-coding exons in a genome. Although the implementation of WES in the diagnostic process improved the diagnostic yield of Mendelian disorders to ∼25–30% (Yang et al., 2013), still many cases of MCDs remain unexplained (de Wit et al., 2008). This holds true even for cases where multiple affected individuals are found in the same family, or other environmental causes have been excluded, strongly hinting at a genetic cause. Even though the diagnostic yield for some MCD groups displaying very defined features on brain imaging, such as lissencephaly (characterized by a smooth brain surface with absent gyri), can reach up to 80% (Di Donato et al., 2018), for the vast majority of cases of the remaining MCD spectrum the yield is much lower (Wiszniewski et al., 2018). Cases displaying this “missing heritability” are often reasoned to be caused by somatic mutations (Jamuar et al., 2014; Gonzalez-Moron et al., 2017), mosaicism (McMahon et al., 2015; Mirzaa et al., 2016; Zillhardt et al., 2016) or by non-genetic causes, such as viral infections (Bosnjak et al., 2011; Moore et al., 2017). However, as WES only interrogates the 1–2% of the human genome that encodes for proteins (Consortium, 2012), it is tempting to speculate that at least some of this missing heritability of MCD might be caused or influenced by genetic variation in the non-coding genome. This hypothesis is supported by several arguments. First, genome-wide association (GWAS) studies on multiple diseases have shown that more than 90% of disease-associated single nucleotide polymorphisms (SNPs) are located outside of coding genes (Maurano et al., 2012), therefore potentially in regions involved in transcriptional regulation. Second, the last decade has witnessed an enormous progress in our understanding of mechanisms involved in gene regulation that find their origin in the non-coding genome, and it has become clear that aberrant gene regulation can cause a variety of genetic disorders (Zeng et al., 2015; Smith and Flodman, 2018; Spielmann et al., 2018). Key elements in the non-coding genome such as promoters, insulators and enhancers, the latter also referred to as non-coding regulatory elements (NCREs), ensure that genes are turned on or off at the right moment in time. When this tight spatio-temporal regulation is disturbed, gene expression can be affected which could result in a genetic disorder. Although only very few large-scale genetic studies have investigated the role of the non-coding genome in genetic disorders (Doan et al., 2016; Devanna et al., 2018; Short et al., 2018) it is clear from the number of excellent studies that have recently been published (Lettice et al., 2002, 2003; Benko et al., 2009; Smemo et al., 2012; Weedon et al., 2014; Ngcungcu et al., 2017; Protas et al., 2017; Bouman et al., 2018; Gloss and Dinger, 2018; Mehrjouy et al., 2018; Potuijt et al., 2018), that it will only be a matter of time until the community fully realizes the importance of the non-coding genome in health and disease. Finally, one and the same mutation can show different degrees of severity in different patients, and it is likely that this phenotypic variability could be influenced by genetic variations outside of coding genes influencing gene expression (Castel et al., 2018; Niemi et al., 2018).

In this *Review*, we will first provide a concise overview of the current state-of-the-art of the field of gene regulation and the role herein of the non-coding genome, with a particular focus on enhancers. We will review recently developed technology and computational approaches that will facilitate future investigations on non-coding causes of MCDs and will discuss examples of non-coding alterations causing genetic diseases. We conclude this *Review* by providing an example of a strategy to identify regulatory alterations in patients with MCDs. Together, this will provide a perspective on how “missing heritability” will be identified in the near future and how to move beyond the borders of the exome.

## The Non-Coding Genome and Its Role in Gene Regulation

According to the central dogma of molecular biology, there are three main processes taking place in the cell: replication of the genetic information, transcription of DNA into RNA, and translation of the RNA molecule into the final functional product, the protein (Crick, 1958). As one of the main surprises from the Human Genome Project, it is now well-established that more than 98% of the human genome does not encode proteins (Consortium, 2012). These non-protein-coding regions were initially considered as junk DNA, which was assumed to be redundant and under no selective pressure, thus allowing for the accumulation of mutations without any harm to the organism (Ohno, 1972; Kuska, 1998). However, several structural elements of non-coding DNA have now been described that regulate gene expression, by determining the 3D genomic organization critical for correct gene regulation. Regulation of gene transcription is particularly crucial during embryonic development, when a single cell needs to differentiate into distinct cell types and to establish diverse gene expression programs in order to acquire a broad range of phenotypes, while maintaining the same genotype. This is achieved by a tight spatio-temporal regulation of gene expression, that allows the transcription of the right gene, at the right level, in the right cell type, and is executed by the interplay between enhancers and gene promoters confined to the “playfield” established by the 3D organization of the genome. It is important to keep in mind in the following paragraphs, that gene regulation, unlike coding DNA, needs to be seen from a non-linear, 3D perspective where regulatory elements need to interact with target genes on long distances.

**FIGURE 1:**
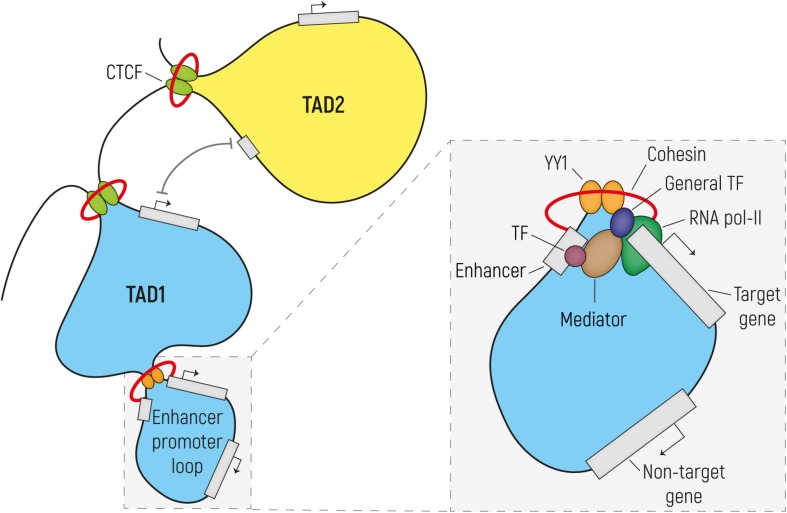
Regulatory enhancer-promoter interactions are restricted within the topological associating domain (TAD) region. The genome (here represented as a black line) is tightly packaged and organized in TADs established by the binding of CTCF to insulator elements, followed by dimerization and interaction with the cohesin complex. In order to establish the enhancer-promoter loops required for transcriptional regulation, enhancers and their target gene should reside in the same TAD. These regulatory loops are formed by the dimerization of YY1 and its interaction with cohesin. In the enlargement is a simplified scheme of transcription initiation (the size does not reflect the actual dimension of each component). Transcription factors (TFs) bind on the enhancer element while the pre-initiation complex formed by the RNA Pol II and the general TFs assembles at the promoter region. Mediator establishes the connection between enhancer and promoter via interactions with TF and components of the transcription preinitiation complex, without binding to DNA. Mediator regulates the phosphorylation of the RNA Pol II in order to release it from the promoter and start transcription.

Genomic organization comprises efficient DNA packaging in the limited space of the nucleus while allowing for DNA replication and gene expression. First, nucleosomes are formed, in which 147 bp of DNA are wrapped around eight histone proteins linked to each other by DNA stretches of various lengths. This beads-on-a-string organization forms the basis of a 10-nm chromatin fiber that is typical of open chromatin, also known as euchromatin. This differs from tightly packaged heterochromatin, where multiple histones wrap into a 30-nm fiber consisting of nucleosome arrays in their most compact form. As a result of this, chromatin is organized into active and inactive compartments that are either open or condensed and which vary in size between 1 to 10 megabases (Mb). Inactive compartments are often found in association with the nuclear lamina, whereas active compartments are more likely to be found in other regions of the nucleus (van Steensel and Belmont, 2017). Regulatory elements such as enhancers and promoters and actively transcribed genes are located in open-chromatin regions, so that they are accessible for the transcriptional machinery. Various post-translational epigenetic modifications of histones put in place by chromatin modifying enzymes can alter the accessibility of chromatin and can thereby influence how chromatin is packaged and whether it is more or less likely to be active. For example, histone acetylation results in increased chromatin accessibility and makes chromatin more available for the binding of regulatory proteins, such as transcription factors (TFs). Many studies focused on a wide variety of histone modifications (see Bannister and Kouzarides, 2011; Hyun et al., 2017, for *Review*), and have led to a draft of a histone code, where various histone modifications are indicative of the functional role that the chromatin has at those places that are modified. For example, putative enhancers are enriched in chromatin regions surrounded by histone 3 lysine 4 monomethylation (H3K4me1) and lysine 27 acetylation (H3K27ac), while promoters are marked by histone 3 lysine 4 trimethylation (H3K4me3). Insulators are responsible for organizing chromatin at a sub-compartment level. They are often bound by the TF CTCF (also known as 11-zinc finger protein or CCCTC-binding factor**)** (Bell et al., 1999) and establish the boundaries of so-called topologically associating domains (TADs). TADs are usually <1 Mb in size and delineate those regions of our chromosomes in which sequences interact preferentially with each-other rather than with elements in other regions of the genome. The prevailing model is that these TADs are formed by the dimerization of two CTCF molecules binding the boundaries of a TAD and are stabilized by the interaction with the ring-shaped cohesin complex through a process called loop extrusion (Dowen et al., 2014; Ong and Corces, 2014; Rowley and Corces, 2018). Inside TADs, smaller DNA loops are formed to allow enhancer–promoter interactions and thus regulation of transcription (Dowen et al., 2014; Hnisz et al., 2016). These enhancer-promoter loops, similarly to the CTCF-mediated loops, are thought to be established by the binding and dimerization of the TF YY1 and its interaction with the cohesin complex (Figure 1) (Beagan et al., 2017; Weintraub et al., 2017).

Promoters are located around the transcriptional start site (TSS) of genes and are essential to initiate transcription. Enhancers are positive regulators of transcription (Banerji et al., 1981), whose location relative to the TSS of the gene they control varies from adjacent to the promoter, to many kilobases (kb) upstream or downstream (e.g., *in cis*), and can even be located in introns, also of other genes. Moreover, besides acting in a position-independent manner, enhancers can regulate transcription irrespective of their orientation. A classic example of a long-range regulatory element is the limb *SHH* enhancer, which is located ∼1 Mb away from its target gene (Lettice et al., 2003). Finally, one enhancer can regulate several genes, and at the same time each gene can be regulated by multiple enhancers. This creates a redundancy in the system that results in phenotypic robustness, and probably gives advantages during evolution (Osterwalder et al., 2018). Therefore, the positions, identities, and arrangements of *cis*-regulatory sequences ultimately determine the time and place that each gene is transcribed. On a mechanistic level, enhancers directly influence the recruitment of the transcriptional machinery to the TSS of genes (Stadhouders et al., 2012; Coulon et al., 2013). Crucial for this long-range control of gene expression by enhancers is the formation of enhancer-promoter loops which preferentially occur within the neighborhood of a TAD, by DNA bending. The general TFs and the RNA polymerase II bind to the promoter sequence, whereas the distal *cis*-regulatory sequences are bound by TFs, which orchestrate the rate of transcription initiation. Enhancers include TF binding sites (TFBSs) that typically consist of DNA motifs found at multiple sites in the genome, but that are not necessarily all equally likely to be bound by the recognizing TF (Lambert et al., 2018). To provide higher than background activity, homotypic or heterotypic dimerization of transcription regulators increases their DNA binding affinity and specificity (Funnell and Crossley, 2012). TF binding itself can also be influenced by DNA methylation, which is established by DNA methyltransferases (DNMTs) (Yin et al., 2017). Moreover, if TF binding prevents the DNA from rewrapping around the nucleosome, it increases the likelihood that a second transcription regulator binds the DNA, increasing the cooperative effect to the extent of displacing the histone core of the nucleosome (Iwafuchi-Doi et al., 2016; Iwafuchi-Doi, 2019). Multiple TFs have been found to bind in a cooperative manner in TF binding site “hotspots” (Siersbaek et al., 2011), later called stretch enhancers (Parker et al., 2013) or super-enhancers (SEs) (Whyte et al., 2013). The latter are described as long regions with an increased density of enhancer elements characterized by a strong enrichment of H3K27ac, and of TFs and Mediator binding (Hnisz et al., 2013; Whyte et al., 2013). On the one hand, a number of studies suggests that SEs represent a novel class of NCREs that maintain, define, and control mammalian cell identity and whose transcriptional regulatory output is larger than that of the individual enhancer constituents (Young, 2011; Whyte et al., 2012, 2013; Loven et al., 2013). On the other hand, an increasing number of studies have challenged this view and consider super enhancers as a collection of normal enhancers that together do not have a larger activity than the sum of the individual parts (Hay et al., 2016; Shin et al., 2016). Therefore, the debate on whether SE are a new class of NCREs or whether they simply reflect a clustering of normal NCREs within close proximity remains to be settled.

What is clear from the above, is that our knowledge on complex gene regulatory mechanisms has increased dramatically over the last decade and has provided insights into many sophisticated processes that need to occur correctly for development to proceed normally. Aberrations in many of the steps described above can result in genetic disorders. For example, in recent years a large number of disorders have been described that are caused by mutations in chromatin modifying enzymes or proteins involved in 3D chromatin regulation (Gregor et al., 2013; Ball et al., 2014; Mirabella et al., 2016; Holsten et al., 2018). Given the complexity of gene regulation and the many contributing factors acting at different stages of this process, it seems likely that many more will be discovered in the near future.

## Genome-Wide Identification of Putative Enhancers

**FIGURE 2:**
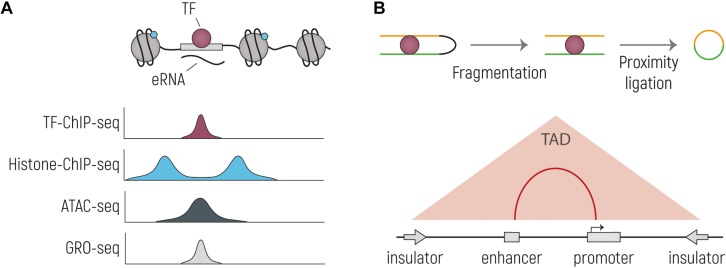
Overview of the main techniques currently used to identify putative enhancer sequences and their interacting genes. **(A)** Schematic drawing of an TF-bound enhancer, located in nucleosome depleted DNA from which eRNA is transcribed. Below are representative genome browser tracks shown, illustrating expected profiles for the same genetic region. Histone-ChIP-seq is illustrative for marks such as H3K27ac and H3K4me1. **(B)** Cartoon representing the main steps of the workflow of Chromosome conformation capture technologies: nuclei are cross-linked, chromatin is then digested and re-ligated by proximity ligation. The two stretches of DNA that are normally located far away from each other (yellow and green), are now ligated together and can be tested by PCR or sequencing. In the bottom part is indicated the output of the experiment, with which TADs and enhancer-promoter interactions can be identified.

As introduced in the previous paragraph, transcriptional enhancers were first described as DNA sequences that are able to enhance gene expression on an episomal plasmid (e.g., a non-integrating, extra chromosomal circular DNA), irrespective of their location and orientation relative to the TSS (Banerji et al., 1981; Moreau et al., 1981); thus, enhancer identification was first limited to low-throughput reporter assays, where small fragments of DNA were tested for regulatory activity influencing reporter gene expression. The most widely applied experimental techniques for genome-wide identification of putative enhancers at the endogenous genomic locus today do not rely directly on this functional property, but rather on features that distinguish enhancers from non-regulatory regions at the chromatin level. Indeed, enhancers are bound by TFs and transcription coactivators and are located in open chromatin regions that are depleted from nucleosomes. The surrounding nucleosomes have specific histone tail modifications, such as the previously mentioned H3K4me1 and H3K27ac. Moreover, some enhancers are bi-directionally transcribed in so-called enhancer RNAs (eRNAs). However, even though these features correlate with enhancers, other genomic regions share the same chromatin characteristics, and more functional tests are required to prove that putative enhancers are indeed having a direct functional role in gene regulation (Catarino and Stark, 2018). This led to the development of high-throughput functional screenings, overall known as massively parallel reporter assays (MPRAs) that quantify the enhancer activity of millions of sequences. In the next paragraphs we will discuss the most widely used techniques to identify putative regulatory regions (Figure 2 and Table 1), and in the following section we will focus on high-throughput functional screens.

**TABLE 1:** Methods for the identification of non-coding regulatory elements (NCRE).

Method	**Description**	**Advantages**	**Disadvantages**
ChIP-seq	Chromatin immunoprecipitation of histone- modifications or TFs coupled with NGS.	Determines genome-wide binding patterns of protein of interest	Not all enhancers are marked by H3K27ac or H3K4me1, or tested TFs. Requires availability of ChIP-grade antibodies. Cannot determine enhancer activity. Cannot identify target gene.
ATAC-seq	Identification of open chromatin regions by the transposon Tn5, that cuts the DNA and inserts sequencing adapters.	Fast. Requires a low number of cells. No need for any *a priori* knowledge.	Other elements are located in open chromatin regions. Cannot determine enhancer activity. Cannot identify target gene.
eRNA detection	Detection of the bidirectionally transcribed eRNA by sequencing the nascent RNA through techniques such as GRO-seq or CAGE.	Identifies enhancer transcription	Not all active enhancers are transcribed.
Chromosome conformation capture	Detection of topological interactions between two loci (3C) or genome wide (4C, 5C, Hi-C).	Identifies enhancer-target gene interactions	Cannot determine enhancer activity.
STARR-seq	Identification of functional enhancers by a massively parallel reporter assay where active enhancers drive their own transcription.	Identifies functional enhancers. Quantitatively measures enhancer activity. High-throughput.	Episomal. Highly complex plasmid libraries requiring substantial number of cells for transfection. Possible false negative results.
CRISPR-Cas9 screenings	Endogenous manipulation of enhancers to force their activation or inactivation.	Identifies functional enhancers. Can be high-throughput. Determines the endogenous effect of enhancer manipulation.	Off-target activity. Possible false negative results.

### Chromatin Immunoprecipitation

Chromatin immunoprecipitation (ChIP) was first introduced more than 30 years ago to study protein-DNA interactions (Solomon et al., 1988) and it follows three basic steps. First, proteins are covalently cross-linked to their DNA binding-site by treating cells with formaldehyde. Chromatin is then sheared, and protein-DNA complexes are selectively co-immunoprecipitated with an antibody against the protein of interest. Finally, the cross-linking is reversed, and DNA is isolated and tested to identify the binding sites of the protein of interest. In more recent years, the emergence of next generation sequencing (NGS) technologies, allowed genome-wide mapping of these protein-DNA binding sites (ChIP-seq) (Johnson et al., 2007; Robertson et al., 2007). ChIP-seq is now primarily used to identify putative enhancers across the entire genome by immunoprecipitation of TFs, specific histone-tail post-translational modifications, including H3K4me1 (Heintzman et al., 2007) and H3K27ac (Creyghton et al., 2010), and transcriptional coactivators, such as the histone acetyltransferase p300/CBP (Visel et al., 2009) and Mediator (Whyte et al., 2013). However, neither the binding of a TF nor the presence of histone modifications provide definitive evidence that a sequence acts as a transcriptional enhancer. For example, tissue specific enhancers can have a certain degree of H3K27ac enrichment in tissues where they are not active (Cotney et al., 2012) and not all H3K27ac marked DNA sequences show enhancer activity when functionality tested (Barakat et al., 2018). Several studies have used ChIP-seq for histone modifications to predict enhancers during human brain development (Reilly et al., 2015; Amiri et al., 2018) and in adult brain (Vermunt et al., 2014; Sun et al., 2016; Li et al., 2018; Wang D. et al., 2018), and some have made direct comparisons to brains from other primates, providing important insights in the evolution of humans (Reilly et al., 2015; Vermunt et al., 2016).

### Identification of Open Chromatin Regions

As abovementioned, *cis*-regulatory sequences like enhancers are enriched in chromatin regions depleted from nucleosomes (Boyle et al., 2008), as nucleosomes would impede TF binding (Lidor Nili et al., 2010). These accessible DNA regions can be identified in a genome-wide fashion thanks to several techniques such as DNase-seq, FAIRE-seq, and ATAC-seq. DNase-seq takes advantage of the hypersensitivity of open chromatin to nuclease digestion. Briefly, cell nuclei are isolated, and DNA is digested with limiting concentrations of DNase I. Fragments of about 500 bp are then selected and used for library preparation and sequencing (John et al., 2013). FAIRE-seq (Formaldehyde-Assisted Isolation of Regulatory Elements) is based on the separation of free and nucleosome-bound DNA. Chromatin is cross-linked with formaldehyde to covalently bind nucleosomes to the DNA, and then sonicated and purified by phenol-chloroform extraction. Nucleosome-bound DNA is sequestered to the interphase, while accessible DNA can be recovered from the aqueous phase and sequenced (Giresi and Lieb, 2009). Finally, the most recently developed method ATAC-seq (Assay for Transposase Accessible Chromatin with high-throughput sequencing) exploits the preference of transposons to land in open chromatin regions. Shortly, the transposon Tn5, loaded with sequencing adapters, is able to simultaneously cut the DNA and insert the adapters in a process known as tagmentation. The open chromatin regions where the transposon preferentially inserts are then amplified with primers binding to the adapters and sequenced. Compared to DNase-seq and FAIRE-seq, ATAC-seq is a simple and fast method that requires less starting material and does not require gel-purification or crosslinking reversal steps and is therefore less prone to loss of material (Buenrostro et al., 2013). However, as mentioned earlier, other regulatory elements such as insulators or promoters are also located in accessible chromatin (Boyle et al., 2008). Therefore, ATAC-seq should be used in combination with other techniques that are more selective for enhancers. Moreover, these methods qualitatively identify putative enhancers and do not allow the quantification of their activity; indeed, also inactive enhancers can be in open-chromatin regions (Arnold et al., 2013; Catarino and Stark, 2018). A major advantage of all techniques assessing chromatin accessibility compared to ChIP-seq is that they screen for putative regulatory regions in an unbiased way, not requiring *a priori* knowledge of enhancer binding factors and not being restricted to the use of available ChIP-grade antibodies. A recent study has used ATAC-seq and RNA-seq to determine open chromatin regions and gene expression at different gestational weeks, and in different areas of the brain, i.e., the ventricular zone and the neuronal layers, providing a first glimpse on open chromatin dynamics during human fetal brain development (de la Torre-Ubieta et al., 2018).

### eRNA

Transcription of enhancer sequences was first reported in the early 90s in the Locus Control Region (LCR) of the β-globin gene cluster (Collis et al., 1990; Tuan et al., 1992; Ashe et al., 1997), where it was found that the expression of the LCR is restricted to the erythroid lineage. Later, transcription of regulatory elements into enhancer RNAs (eRNAs) was validated genome-wide with sequencing, at first, of total neuronal RNA (Kim et al., 2010), followed by sequencing of nascent RNA (GRO-seq, CAGE) in different cell types (Wang et al., 2011; Hah et al., 2013; Kaikkonen et al., 2013; Andersson et al., 2014). Enhancer RNAs are generally bidirectionally transcribed and not polyadenylated (Kim et al., 2010) but reports of unidirectional transcription and polyadenylation of eRNAs exist (Koch et al., 2011). Enhancer transcription was shown to correlate with the presence of other enhancer marks such as histone tail post-translational modifications and p300/CBP and RNApolII binding (Wang et al., 2011; Hah et al., 2013; Kaikkonen et al., 2013), but whether their expression is a cause, or a consequence of gene transcription is still debated (Lam et al., 2014). If eRNA transcription has a direct functional role and is not just noise due to the recruitment of RNApolII, the effect can either be mediated by the transcription process itself or by the transcript produced upon transcription, which might have direct *cis*-regulatory activity similar to other non-coding RNAs such as those involved in X chromosome inactivation (Barakat and Gribnau, 2010; Natoli and Andrau, 2012). However, even if eRNA presence correlates with enhancer activity at some loci, it seems that it is neither required nor sufficient in all instances (Catarino and Stark, 2018). For example, a recent study assessing eRNAs in brain only found that around 600 intergenic and intronic enhancers are transcribed in eRNAs, and this number even further decreased when considering only those eRNAs replicated in an independent data set or overlapping with enhancer associated histone modifications (Yao et al., 2015). The FANTOM project has found a similar small number of eRNAs in brain, although the majority of those are not overlapping with those from Yao and colleagues (Andersson et al., 2014). The number of predicted brain related enhancers based on other assays by far outnumbers this rather small set of transcribed enhancers, indicating that methods that just take eRNA transcription into account may oversimplify the identification of putative enhancers and may not catch the complete regulatory landscape.

### Identification of Long-Distance Chromatin Interactions

All methods described until now identify putative enhancers but understanding which genes they regulate remains a challenge. Indeed, despite often regulating nearby genes, enhancers can also be found at long distances from the TSS of their target gene. Moreover, as abovementioned, it is becoming more and more clear that chromatin organization plays an important role in transcription and, as abovementioned, enhancers and promoters need to be brought in close proximity in order for transcription to take place. In the past ∼20 years several techniques have been developed to address this question (reviewed in de Wit and de Laat, 2012; Davies et al., 2017). The pioneering method, on which all the later developments are based, is known as chromosome conformation capture (3C) and relies on the formaldehyde cross-linking of chromatin within nuclei, followed by restriction digestion of chromatin and re-ligation by proximity ligation. The obtained fragments represent the junction of two chromatin regions that are normally located far away from each other on the linear genome, but are in close proximity in 3D space, and these junction products can be quantified by PCR (Dekker et al., 2002). 3C was developed to study whether two known regions are interacting with each other and is thus described as a “one vs. one” method (de Wit and de Laat, 2012). Further advances of 3C-based techniques allowed the identification of increasing numbers of contacts; for example, 4C, “one vs. all,” allows the identification of all the regions interacting with a specific site of interest (Simonis et al., 2006; Zhao et al., 2006), while 5C, “many vs. many,” investigates all contacts that are happening in a specific locus (Dostie et al., 2006). Finally, high-throughput contact identification became possible with Hi-C (Lieberman-Aiden et al., 2009). Hi-C allows the identification of genome-wide interactions thanks to the introduction of biotin-labeled nucleotides at the sites of restriction-digestion. The ends are then ligated, the chromatin is sheared, and the junctions are enriched by streptavidin pull-down and sequenced. By the application of an algorithm on Hi-C data, TADs can be defined. To investigate all the genome-wide interactions involving a specific protein of interest, HiChIP was developed, by introducing a chromatin immunoprecipitation step (Mumbach et al., 2016). This method has the advantage that it requires less input material and less sequencing reads.

Despite their capacity to identify enhancer-promoter interactions and thereby pieces of chromatin with putative regulatory roles, chromatin conformation techniques have the disadvantage of not directly measuring functional regulatory activity. Moreover, in most cases, interactions are determined on a population level on a high number of cells, which might only provide a snapshot of dynamic regulatory interactions. Finally, the spatial resolution at which interactions can be determined is heavily influenced by the sequencing depth of Hi-C experiments. Hence, there remains a need for more functional tests to validate the regulatory activity of the identified interactions. A recent study has generated Hi-C maps from gestational weeks 15, 16, and 17 of human brain development, a critical time period for cortex development (Won et al., 2016), permitting the large-scale annotation of previously uncharacterized regulatory interactions relevant to the evolution of human cognition and disease. For example, the results of this study have linked several non-coding variants identified in GWAS to genes and pathways involved in schizophrenia, highlighting novel mechanisms underlying neuropsychiatric disorders.

## High-Throughput Functional Identification of Enhancers

**FIGURE 3:**
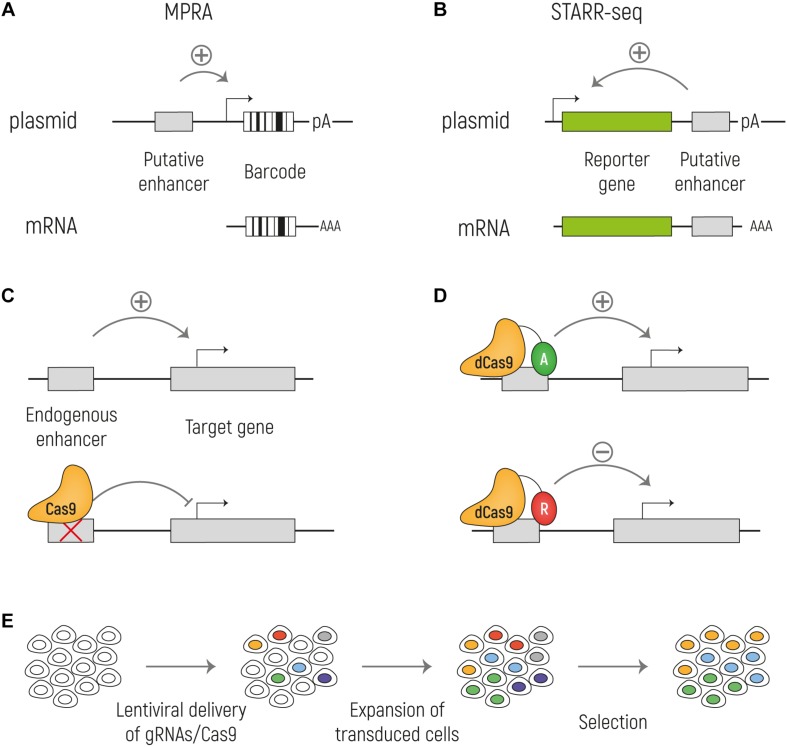
Methods for functional identification of enhancers. **(A)** Massively parallel reporter assays (MPRA) to test enhancer activity in an episomal setup. The putative enhancer sequence is cloned upstream a minimal promoter that drives the expression of a reporter gene and a unique barcode. **(B)** With STARR-seq the putative enhancer sequence is cloned downstream the reporter gene and upstream of the polyA signal. When the enhancer sequence is active, it can drive the expression of the reporter (green) and of itself. In both MPRA and STARR-seq the mRNA is sequenced to identify the active enhancers. **(C)** Cas9 can be used to knock out an enhancer at the endogenous genomic locus to assess its effect on the target gene transcription. **(D)** A catalytically inactive Cas9 (dCas9) can be fused with activators (A: VP64; TET1; p300) or repressors (R: KRAB; SID4X; DNMT3A; KDM1A). **(E)** Cas9 screens can be combined with high-throughput screenings by targeting Cas9 expressing cells with a lentiviral library of gRNA at a low MOI. By doing so, each cell will express a single gRNA and by different selections, such as drug resistance or reporter gene expression, it is possible to investigate the effect of the ablation of a large number of putative enhancers on gene expression in parallel.

As previously highlighted, most of the commonly used techniques to identify regulatory elements are merely predictive, and do not directly measure enhancer activity. Although there is no doubt that techniques such as ChIP-seq, open chromatin mapping and expression analysis have been of tremendous use to globally characterize the gene regulatory landscape of the non-coding genome, it is still clear that there is a need for improved techniques. In many instances, the identified putative enhancer sequences fail to perform as enhancers in functional validation experiments, giving rise to false positive enhancer predictions (Kwasnieski et al., 2014; Halfon, 2019, for an excellent review). Moreover, the resolution of commonly used techniques usually allows the identification of regions in the range of 500–1000 bp as potentially including an enhancer. But this makes it difficult to pinpoint those nucleotides that are of real functional relevance within a given predicted enhancer sequence, and this complicates, for example, the assignment of functional roles of nucleotide variants found in the human population. Finally, many of the currently used techniques take into consideration previously identified knowledge on associations between epigenetic marks and putative enhancers. This potentially excludes other regions of the genome to be functionally assessed as they lack these associations but might nevertheless be functionally relevant (Pradeepa et al., 2016). Direct high-throughput functional tests of enhancer activity, such as massively-parallel reporter assays (MRPAs) and CRISPR-Cas9 based screens have the potential to address these shortcomings (Figure 3), as we will explain in the next section.

Most traditional functional tests for enhancer activity are based on reporter assays, in which a putative enhancer sequence is cloned into a vector with a reporter gene driven by a minimal promoter that alone is not sufficient to induce reporter gene expression. The vectors are then transfected into a cell line or organism of interest, and the reporter gene expression is determined (Banerji et al., 1981). MPRAs are high throughput reporter assays where DNA sequences are inserted before the minimal promoter of a vector with a specific barcode sequence downstream of the open reading frame, which allows the simultaneous assessment of 1000s of sequences for enhancer activity in parallel (Melnikov et al., 2012; Patwardhan et al., 2012; Arnold et al., 2013; Kheradpour et al., 2013; Whyte et al., 2013; Dickel et al., 2014; Murtha et al., 2014; Ernst et al., 2016). After cell transfection, RNA can be purified and sequenced. If the sequence cloned into the vector is a functional enhancer it drives the expression of the corresponding barcode. An adapted approach is Self-Transcribing Active Regulatory Region (STARR) sequencing (Arnold et al., 2013). STARR-seq takes advantage of the fact that enhancers act in a position-independent fashion. Indeed, differently from other MPRAs, STARR-seq does not rely on barcodes, but the candidate sequences are cloned downstream of the TSS and, when active, drive their own transcription. With this assay, millions of sequences can be tested in a single experiment. In both cases, the activity of the enhancer can be measured by the relative abundance of the barcode/sequence transcript from RNA-seq, in comparison to sequencing of the input plasmids. Similar episomal high-throughput approaches have recently been developed to also measure promoter responsiveness to enhancers (Arnold et al., 2017) and autonomous promoter activity (van Arensbergen et al., 2017).

The major advantage of these tests is that they are unbiased, since they are not based on any *a priori* hypothesis about TF binding or histone modifications. Nevertheless, the size of the human genome requires the construction and transfection of large plasmid libraries, and thus substantial numbers of cells and deeper sequencing and might therefore lead to a lower resolution. To overcome this limitation, it is possible to focus STARR-seq only on putative enhancers, testing only the sequences identified with ChIP (Barakat et al., 2018), ATAC (Wang X. et al., 2018), or other techniques (Vanhille et al., 2015; Shen et al., 2016). In our own application, we have combined ChIP with STARR-seq to generate genome-wide enhancer activity maps in various types of human embryonic stem cells (Barakat et al., 2018).

Despite being incredibly useful to test millions of sequences for enhancer activity in a high-throughput manner, reporter gene assays may have several limitations. First, enhancer activity is tested most often on an episomal background, which might not completely reflect endogenous gene regulation in its native genomic context (Inoue et al., 2017). Interestingly, recent studies suggest that the effects of this might be less strong than initially suggested, as there is a high correlation between episomal enhancer activity and endogenous gene regulation when assessed by CRISPR-based deletions (Barakat et al., 2018), or when a set of enhancers is assessed on both plasmids and integrated at multiple genomic locations (Maricque et al., 2018). Second, MPRAs may potentially give false negative results. Indeed, if a sequence is found inactive in a reporter assay, this does not exclude that it is active as an enhancer in a different cell type, in a different moment in time or has another, but still biologically relevant, role independent on enhancer activity (Shlyueva et al., 2014).

One way to overcome these possible limitations of transgenic reporter assays, is to use the recently developed CRISPR-Cas9 system to manipulate NCREs at the endogenous chromatin context. Cas9 is an RNA-guided DNA endonuclease that is able to induce double-strand breaks that, in the absence of a donor template for homology directed repair, are repaired by the error-prone non-homologous end-joining (NHEJ). The enhancer sequence can thus be deleted, by targeting Cas9 with guide-RNAs (gRNAs) flanking the enhancer sequence or be mutated by the introduction of indels via NHEJ, allowing to test the effect of the enhancer ablation on gene expression in the endogenous chromatin environment. Whereas this approach can be used to study a selected enhancer of interest, as we have done studying enhancers involved in pluripotency of human embryonic stem cells (Barakat et al., 2018), it can also be used in high-throughput screenings with large libraries of gRNAs that are introduced in cells expressing Cas9. Lentiviral transduction of gRNAs at a low multiplicity of infection can result in a single gRNA integration per cell, and in combination with various means of positive or negative selection, such as drug selection or assessment of reporter gene expression, this can be used to investigate in parallel and on a large scale the effect of multiple putative enhancer ablations on gene expression. To this end, large populations of cells are transduced, and the quantitative presence of gRNAs is determined by next generation sequencing of isolated DNA prior and after a selection. If a sequence has an important role in gene regulation, the ablation of that sequence is expected to result in disadvantage for the cells, and therefore gRNAs targeting relevant functional NCREs will be depleted over time. By comparing sequencing reads after and prior to the selection, it is possible to determine which gRNAs are lost over time, and as the targets of the gRNAs are known, the relevant NCRE can be identified. In one of the first applications, DNA regions around the *TP53* and *ESR1* gene loci were investigated, and it was shown that this approach was feasible to identify functional enhancers and, furthermore, using a dense CRISPR-Cas9 gRNA tilling screen, functional domain within these enhancer sequences were precisely mapped (Korkmaz et al., 2016). Using a similar approach, more than 18.000 gRNAs were used to test around 700 kb of sequence flanking genes involved in BRAF inhibitor resistance in melanoma, finding non-coding regions involved in gene regulation and chemotherapeutic resistance (Sanjana et al., 2016). Other studies investigated putative enhancers involved in oncogene induced senescence (Han et al., 2018), regulation of the *HPRT* gene involved in Lesch–Nyhan syndrome (Gasperini et al., 2017) and regulation of the *POU5F1* gene in embryonic stem cells (Diao et al., 2016, 2017), amongst others (Canver et al., 2015, 2017; Rajagopal et al., 2016; Sen et al., 2016). Besides genome-engineering, CRISPR-Cas9 can also be applied to edit the epigenome, and also this can be coupled to high-throughput screening. Indeed, by fusing a catalytically dead Cas9 (dCas9), that lacks endonuclease activity, to various functional domains it is possible to alter the status of a NCRE forcing its activation or inactivation, referred to as CRISPRa and CRISPRi, respectively. Functional additions to dCas9 leading to NCRE activation include transcription activating domains such as multiple repeats of the herpes simplex VP16 activation domain (VP64) (Maeder et al., 2013; Mali et al., 2013), the nuclear factor-κB (NF-κB) trans-activating subunit activation domain (p65) and human heat-shock factor 1 (HSF1) (Konermann et al., 2015), the 10–11 translocation methylcytosine dioxygenase 1 (TET1) (Liu X. S. et al., 2016), and the p300 acetyltransferase (Hilton et al., 2015). Opposingly, transcription repressive domains that can be used to silence NCREs include Krüppel-associated box (KRAB) domain (Gilbert et al., 2013; Thakore et al., 2015), four concatenated mSin3 domains (SID4X) (Konermann et al., 2013), cytosine-5-methyltransferase 3A (DNMT3A) (Vojta et al., 2016), Histone deacetylase 3 (HDAC3) (Kwon et al., 2017), and the lysine-specific histone demethylase 1A (KDM1A), called dCas9-LSD1 (Kearns et al., 2015). Several of these dCas9 fusion have been used to activate or repress NCREs, and a number of studies have used them in high-throughput screening approaches, most of which focused on NCRE repression (Fulco et al., 2016; Carleton et al., 2017; Xie et al., 2017; Gasperini et al., 2019) but some included also NCRE activation (Klann et al., 2017; Simeonov et al., 2017). It seems only a matter of time till more similar studies editing NCREs in various cell types using the full CRISPR-Cas9 toolbox will be published. Obviously, as all experimental approaches, also CRISPR-Cas9 has its pitfalls and is still far from perfect. For example, reduced on-target activity and off-target effects of gRNAs can introduce experimental noise, and it remains essential that screening results are validated independently. Also, it remains to be seen whether subtle enhancer effects on gene expression, that might still be of biological relevance, can be detected using CRISPR-based screens.

## Computational Approaches

As it has become clear from the discussion above, currently used enhancer prediction and validation techniques heavily depend on computational data analysis, most often involving the analysis of next generation sequencing data. Besides the direct use of computational analysis for biological data processing, more and more efforts are undertaken to use computational power to predict functional NCREs *in silico*. These methods can be broadly summarized in approaches that rely on (1) comparative genomics and evolutionary conservation, (2) clustering of motifs and epigenome features and machine learning approaches, and (3) techniques that deal with the processing of data obtained in functional genomic screens. Here, we mainly focus on the advantages and disadvantages of some of these methods and highlight several resources that can be used to obtain information on genomic enhancer locations. We refer those readers who are interested in a more detailed discussion on the various options for machine learning and other prediction tools to a number of excellent recent reviews (Wang et al., 2013; Suryamohan and Halfon, 2015; Kleftogiannis et al., 2016; Lim et al., 2018).

### Comparative Genomics and Evolution in Enhancer Prediction

Functional sequences are expected to be more conserved compared to DNA stretches that are not expected to have any role, as changing of nucleotide composition is expected to alter function. This characteristic is exploited by comparative genomics approaches that aim to identify enhancers by looking at the most conserved sequences across different species. This approach was one of the first computational tools to identify NCREs (Pennacchio et al., 2006; Li et al., 2007). Nevertheless, different studies showed how some NCREs are strongly conserved, while others are rapidly changing also in closely related-species, rendering the solely use of comparative genomics techniques insufficient. For example, Arnold et al. (2014) showed by STARR-seq of different *Drosophila* species how, in the majority of the cases, enhancer function is conserved across species, and the highly conserved enhancers are thought to play an important role during key processes such as embryonic development, and especially in the developing nervous system (Pennacchio et al., 2006). However, several other studies suggest that a portion of enhancers undergo rapid evolution, and that this might be a crucial driver of human evolution (Degner et al., 2012; Shibata et al., 2012; Villar et al., 2015; Glinsky and Barakat, 2019). A subset of active enhancers in human embryonic stem cells is even enriched in human specific transposable elements, and those functional regions would be missed if one were to use only conservation as a key feature for enhancer selection (Barakat et al., 2018). Therefore, although sometimes useful, evolutionary conservation alone for the discovery of NCRE is not recommended as a sole criterion, as it would miss all the newly evolved enhancers. Another extreme example of this are so-called ultraconserved elements, stretches of DNA sequences that are more than 200 bp long and that are 100% identical in multiple species, such as human, rat and mouse (Bejerano et al., 2004). Whereas some of these sequences were shown to play a role as enhancers (Visel et al., 2008; Dickel et al., 2018), others can be removed from the genome without an obvious phenotype (Ahituv et al., 2007), and it is speculated that some of these sequences might contribute to genome stability (McCole et al., 2018). Enhancers can also be identified by the presence of specific TFBS, as TF binding is a key characteristic of these regulatory sequences. Indeed, combining conservation with TFBS site discovery can further increase the predictive power of comparative genomic approaches. However, even this does not guarantee enhancer identification, as during evolution novel TFBS can appear which execute similar functions as the ones in the ancestry sequence (Ludwig et al., 2000).

### Enhancer Prediction Algorithms

Several types of enhancer predicting algorithms have been developed for integrating multiple types of data, such as TF motifs, ChIP-seq, DNase-seq, ATAC-seq, and P300 binding data sets for enhancer prediction by using clustering and machine learning approaches. These algorithms include supervised tools that rely on high-confidence positive and negative training sets (e.g., known- and non-enhancers) to build models that can maximize the differentiation between enhancer and non-enhancer sets, and non-supervised tools that are used to identify hidden and unknown patterns directly from data. Examples of supervised algorithms include CSI-ANN (Firpi et al., 2010), ChromaGenSVM (Fernandez and Miranda-Saavedra, 2012), RFECS (Rajagopal et al., 2013), EnhancerFinder (Erwin et al., 2014), DEEP (Kleftogiannis et al., 2015), DELTA (Lu Y. et al., 2015), PEDLA (Liu F. et al., 2016), REPTILE (He et al., 2017), eHMM (Zehnder et al., 2019), and DBN (Bu et al., 2017). Other unsupervised algorithms such as Segway (Hoffman et al., 2012) and ChromHMM (Ernst and Kellis, 2012) integrate multiple types of epigenome data to define chromatin segmentations that can be used to assign functional roles for various parts of chromatin. One of the main problems of all these prediction programs is that we still lack a detailed understanding of the underlying regulatory code in the non-coding genome. Despite all the advances made over the last decade, we are yet to pinpoint a feature that can identify enhancers (and their activity) in all cell types. As most programs rely on previously generated training sets or on static features such as DNA sequence motifs which by their own do not necessarily predict enhancers in each instance, it is more than logical that despite the large amount of efforts that are undertaken, enhancer prediction programs are far from perfect. For example, although chromatin segmentation is very intuitive and access to these segments can be easily obtained from the UCSC genome browser, it is rather worrying that a recent study testing more than 2000 sequences classified as enhancers using these methods did not detect regulatory activity in 74% of the sequences tested (Kwasnieski et al., 2014). Also the overlap between individual predictions from various programs is rather poor (Kleftogiannis et al., 2016). Quite intuitively, programs that take into account multiple features for enhancer prediction tend to perform better (Erwin et al., 2014; Dogan et al., 2015; He et al., 2017). Therefore, it is tempting to speculate that future large-scale meta-analyses of all currently available enhancer data might enable the further fine tuning of enhancer prediction tools in the near future. Another area where further progress needs to be achieved is the prediction of enhancer-promoter interactions, that can be used to assign NCREs to their target genes. Several tools for this are currently available, such as ELMER 2 and InTAD. ELMER 2 computes the correlation between the enhancer and target genes by combining both DNA methylation and gene expression data derived from the same data set (Silva et al., 2018), but is limited by the fact that correlations are restricted to the closest neighboring gene, which does not necessarily present the real biological relevant target gene (Kvon et al., 2014). InTAD is a tool to detect genes located upstream and downstream of the enhancer in the same TAD boundary and it can support different types of data as input. The TAD information comes from available Hi-C datasets (Okonechnikov et al., 2019), but the currently available ones have a low resolutions and, up to date, have include a limited number of cell types. Therefore, to fully identify enhancer-gene interactions, it will be important to come up with novel experimental procedures that will enable us to directly test the biological relevance of enhancer-promoter predictions. Finally, a number of programs are developed that aim to predict the functional relevance and possible pathogenicity of variants in NCREs. These include, amongst others, RegulomeDB (Boyle et al., 2012), HaploReg (Ward and Kellis, 2012), CADD (Kircher et al., 2014), GWAVA (Ritchie et al., 2014), GenoCanyon (Lu Q. et al., 2015), Genomiser (Smedley et al., 2016), and INFERNO (Amlie-Wolf et al., 2018). In addition, a number of databases have been generated, including HEDD (Wang Z. et al., 2018), DiseaseEnhancer (Zhang et al., 2018), and EnDisease (Zeng et al., 2019) which have collected NCREs that are related to diseases based on the current literature. It will be crucial to further expand and curate these collections of disease relevant enhancers in the future, and combine them with improved ways of variant interpretation, to fully exploit the relevance of the non-coding genome in disease.

### Available Enhancer Databases

As the available information on the non-coding genome is increasing rapidly over the last decade, more and more resources are available online that can help to localize NCREs and interpreting their functional roles. In the next part, we summarize a selection of databases and resources that are currently available (Table 2) and can be used to find NCREs of relevance for brain development.

**TABLE 2:** Enhancer databases.

**Database**	**Source**
VISTA	http://genome.lbl.gov/vista/index.shtml
EnhancerAtlas 2.0	http://www.enhanceratlas.org/
FANTOM5	http://slidebase.binf.ku.dk/human_enhancers/presets
PsychENCODE	http://development.psychencode.org/#
dbSUPER	http://asntech.org/dbsuper/

One of the first resources of experimentally tested NCREs was the VISTA enhancer database (Visel et al., 2007). Based on comparative genomics, a large selection of putative NCREs from mouse and human was selected and tested in transgenic mouse embryo assay, to determine their *in vivo* enhancer activity, as determined by LacZ expression. The database provides detailed information on the genomic localization of the tested sequences, likely associated genes, and images of transgenic mouse embryos identifying the localization of enhancer driven LacZ expression. Based on the 2/25/2019 update, this database contains 542 tested enhancers active in human forebrain, hindbrain, and midbrain. In addition, the VISTA tool portal can be used as a comparative tool and users can submit their own sequences to conduct comparison against multiple species (Visel et al., 2007), thereby possibly identifying conserved functional NCREs.

EnhancerAtlas 2.0 is a database that has collected putative NCREs based on publicly available data obtained from ChIP-seq for different histone modification, TFs, EP300, and POLII, CAGE and eRNA expression, interaction studies by ChIA-PET (a method that combines 3C with chromatin immunoprecipitation) and chromatin accessibility as determined by FAIRE and DNase-seq. Each putative enhancer is supported by at least three independent high-throughput data sets although the database does not contain any direct functional validations. It contains more than 4,506,217 putative enhancers from 8,573 datasets of 179 human tissue/cells, which through an interactive website can be easily accessed. The 49,925 human fetal brain and 17,103 cerebellum enhancers were predicted using DNa-seq, CAGE, and H3K4me1, and H3K27ac deposition (Gao et al., 2016).

The FANTOM5 database, is the latest version of the FANTOM project, that aims to generate an atlas of mammalian regulatory elements, transcriptomes, and long-noncoding RNAs. NCREs are predicted from sequencing data from cap analysis of gene expression (CAGE) along with RNA-Seq data from multiple tissue and cell types from different developmental time points (Andersson et al., 2014). In total, the database contains more than 43,000 putative enhancers, of which 639 are expressed in brain and 376 were found in neuronal stem cells.

Recently, the PsychENCODE consortium has released data from a large multi-center effort trying to map NCREs during brain development (Amiri et al., 2018; Li et al., 2018; Wang D. et al., 2018). Using analysis of transcriptome, methylation status, histone modifications and even single cell/nucleus-level (transcriptome) genomic data, NCREs were discovered across multiple brain regions over the entire span of human neurodevelopment and from adult brains, and an integrative data analysis was performed. These data, generated from age- and often donor-matched samples, represent the most comprehensive multi-platform functional genomic analysis of the developing human brain performed so far. The analysis resulted in 79,056 enhancers identified from adult brains enriched for H3K27ac and depleted for H3K4me3 (Wang D. et al., 2018). In addition, 96,375 enhancers were shown to interact with protein coding genes during fetal brain development and during *in vitro* differentiation of brain organoids (Amiri et al., 2018). Of the latter, 46,735 enhancers were active only in fetal cortex.

Several super-enhancer databases have been generated such as dbSUPER (Khan and Zhang, 2016), SEA (Wei et al., 2016), and SEdb (Jiang et al., 2019) providing annotation, genomic coordinates and length of super-enhancers, and their possible associated genes. Among those, dbSUPER is one of the most popular databases with 82,234 super-enhancers from multiple human and mouse cell types. In this database, there are 6,002 and 1,114 super-enhancers detected by H3K27ac enrichment from seven human and three mouse brain tissues and cell types, respectively (Khan and Zhang, 2016).

Finally, GeneHancer is a database of human enhancers and their inferred target genes (Fishilevich et al., 2017). Integrating enhancer predictions from ENCODE, Ensembl, FANTOM and VISTA yielded more than 280,000 candidate regions that where assigned to their target genes based on co-expression correlation, expression of quantitative trait loci and capture Hi-C.

**FIGURE 4:**
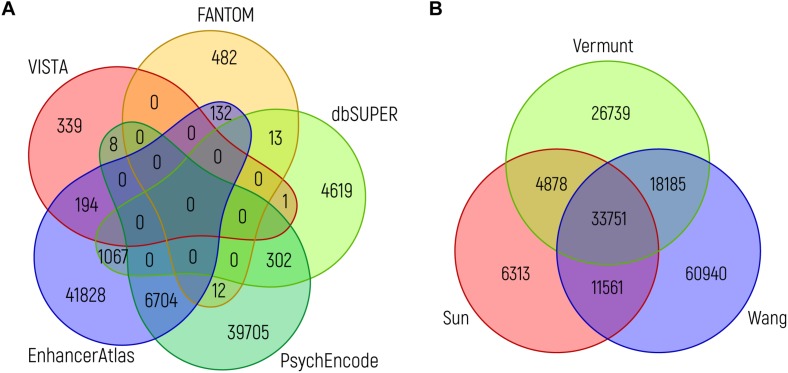
Overlap between brain enhancer databases. **(A)** Venn diagram showing the overlap between brain enhancers (in genome build hg19) from different databases: VISTA (*n* = 542) (Visel et al., 2007), dbSUPER (*n* = 6002) (Khan and Zhang, 2016), FANTOM5 (*n* = 639) (Andersson et al., 2014), PsychENCODE (*n* = 46731; the 46735 enhancers mentioned in the text are in genome build hg38 and the difference of four loci is due to liftover to hg19) (Amiri et al., 2018) and EnhancerAtlas 2.0 (*n* = 49925) (Gao et al., 2016). **(B)** Venn diagram showing the overlap between ChIP-seq peaks for H3K27ac from adult brain, as identified in three studies: Sun (*n* = 56503) (Sun et al., 2016), Vermunt (*n* = 83553) (Vermunt et al., 2014), and Wang (*n* = 124437) (Wang D. et al., 2018). The intersection between different sources (in the same order as above) was performed using bedops and bedtools. In both graphs, the minimum overlap of a single nucleotide is required.

Although all of these databases can easily be accessed and are user friendly, it is important to realize when using them that it is still difficult to judge which of the sources provides the user with those sequences that are indeed most likely to be of functional biological relevance. To illustrate this, it is interesting to compare the overlap between predicted enhancers from the various resources. When we compare putative brain enhancers from VISTA, EnhancerAtlas 2.0, FANTOM5, PsychENCODE and dbSUPER, the overlap between the various enhancer predictions is rather limited, even when considering a single nucleotide as the required overlap (Figure 4A). The same holds true when assessing the overlap between ChIP-seq peaks for H3K27ac from key adult brain related data sets (Figure 4B). Intuitively, one would expect that those NCREs that are found in multiple data sets are more likely to have a true biological role, although this might be an oversimplification, as the brain is a very heterogeneous organ with many different cell types that might differ in NCRE landscape, and technical limitations might still hinder us from detecting all relevant NCREs in each cell type. Ideally, future studies should generate genome-wide functional activity maps of NCREs for all cell types during brain development. A challenge that is probably easier to address on paper than in practice in the near future.

## Aberrations of Non-Coding Elements in Neurodevelopmental Disorders and Malformations of Cortical Development

As previously discussed, an increasing number of studies suggests that a high fraction of causative mutations in neurodevelopmental disorders such as intellectual disability and autism, belong to pathways of transcriptional regulation and chromatin remodeling (Gregor et al., 2013; De Rubeis et al., 2014; Gabriele et al., 2017). Besides mutations in *trans-* acting factors such as TFs or chromatin modifiers, also mutations of NCREs *in cis* have been proven to be causative of disease in an increasing number of cases. A classic example is pre-axial polydactyly caused by alterations of the ZRS, a long-distance enhancer that regulates Sonic hedgehog (*SHH*) expression in the embryonic limb (Lettice et al., 2002; Gurnett et al., 2007). Next to point mutations, also copy number variation (CNVs) such as duplications (Klopocki et al., 2008), and insertions (Laurell et al., 2012) in this region have all been shown to cause polydactyly phenotypes, illustrating the wide range of alterations that can affect enhancer function and thereby result in a phenotype. In this section, we discuss a number of other examples of NCRE alterations, mainly in relation to disorders affecting the brain (Table 3).

**TABLE 3:** Alterations of non-coding regulatory elements in diseases related to the central nervous system.

Disease	**Mutation**	**Affected gene**	**References**
Holoprosencephaly	Point mutation	*SHH*	Jeong et al., 2008
Aniridia	Point mutation	*PAX6*	Bhatia et al., 2013
Polymicrogiria in the Sylvian fissure	Deletion	*GPR56*	Bae et al., 2014
Parkinson’s disease	SNP	*SNCA*	Soldner et al., 2016
Schizophrenia	Tandem duplications	*VIPR2*	Vacic et al., 2011
Adult-onset demyelinating leukodystrophy	Deletion of TAD boundary and deletions	*LMNB1*	Giorgio et al., 2015; Nmezi et al., 2019
Intellectual disability	CNV	*ARX*	Ishibashi et al., 2015

Holoprosencephaly, a neurodevelopmental disorder characterized by craniofacial malformations, can be caused by coding mutations in the *SHH* gene. However, a point mutation in the *SHH* Brain Enhancer 2 (SBE2) was identified, located 460 kb upstream of the *SHH* gene in a patient with an identical phenotype (Jeong et al., 2008). This mutation was found to be disease-causing, as it disrupts the binding site of the TF SIX3, thereby leading to reduced forebrain SHH expression. In agreement, also mutations in *SIX3* can lead to holoprosencephaly (Wallis et al., 1999). A disease-causing enhancer mutation is also found in the congenital eye malformation aniridia, that is often caused by haploinsufficiency of the TF PAX6, that also plays crucial roles in neural stem cells. A point mutation in the *PAX6* eye-enhancer was found to disrupt PAX6 binding, thereby affecting *PAX6* expression (Bhatia et al., 2013). In another example, a 15-base pair deletion in a regulatory element upstream of an alternative transcript of *GPR56* was found in five individuals from three families (Bae et al., 2014). *GPR56*, when mutant in its coding sequence, leads to widespread cobblestone malformation with cerebellar and white matter abnormalities. In the patients carrying the 15-bp regulatory element deletion, polymicrogyria was bilaterally restricted to the Sylvian fissure, leading to a phenotype of speech delay, intellectual disability and refractory seizures without further motor involvement. The authors could show that the deletion disrupts an RFX binding site, and thereby specifically alters the expression of *GPR56* in the perisylvian and lateral cortex, including the Broca area that is the primary language area.

Besides influencing disorders presenting early in life, also disease emerging later in life, such as neurodegenerative disorders and schizophrenia, are increasingly linked to variants in NCREs. For example, a risk variant in an enhancer regulating α-synuclein expression was recently shown to affect gene expression by altering the binding of the TF EMX2 and NKX6-1 (Soldner et al., 2016). In addition, tandem duplications of the non-coding upstream region of *VIPR2* have been observed in cases of schizophrenia and resulted in upregulated *VIPR2* expression (Vacic et al., 2011). Also, CNVs overlapping with NCREs in other schizophrenia related genes might be implicated in the disease pathogenesis, influencing the disease vulnerability (Piluso et al., 2019).

Multiple CNVs have also been associated with periventricular nodular heterotopia (PNH), a brain malformation in which nodules of neurons are ectopically retained along the lateral ventricles (Cellini et al., 2019). Besides changing gene dosage, CNVs can also change the dosage and position of NCREs, as well as the higher-order chromatin organization of a locus (Feuk et al., 2006; Spielmann et al., 2018). Similarly, copy-number-neutral structural variants, such as inversions and translocations, can disrupt coding sequences or create fusion transcripts, but these types of variants can also disrupt or create new enhancer landscapes and chromatin domains, resulting in regulatory loss or gain of function. A clinical example of such a structural variant that changes the 3D architecture of the genome is the deletion of a TAD boundary at the *LMNB1* locus, which causes an enhancer to regulate a gene that is normally not regulated by that enhancer (so-called enhancer adoption). In this case, the enhancer adoption leads to adult-onset demyelinating leukodystrophy (ADLD), which is a progressive neurologic disorder affecting the myelination of the central nervous system (Giorgio et al., 2015). More recently, deletions upstream of *LMNB1*, varying in size from 250 to 670 kb, occurring in repetitive elements, have revealed increased LMNB1 expression and an atypical ADLD phenotype (Nmezi et al., 2019). Other rare inherited structural variants in *cis*-regulatory elements might influence the risk for children of developing autism spectrum disorders (ASDs), depending on the parental origin of the structural variant (Brandler et al., 2018). Another study on autism using whole genome sequencing (WGS) on more than 2000 individuals found that probands carry more gene-disruptive CNVs and SNVs resulting in severe missense mutations and mapping to predicted fetal brain promoters and embryonic stem cell enhancers (Turner et al., 2017). In addition, CNVs covering the regulatory elements of the *ARX* gene might cause an intellectual disability phenotype (Ishibashi et al., 2015), and rare non-coding CNVs near previously known epilepsy genes were enriched in a cohort of 198 individuals affected with epilepsy compared to controls (Monlong et al., 2018). Similar findings are reported for multiple system atrophy (Hama et al., 2017) and non-coding variants might influence expression of *GLUT1* causing epilepsy (Liu Y.C. et al., 2016).

Two large-scale analyses focused on NCREs and their role in neurodevelopmental disorders have recently been performed. Using a targeted sequencing approach, Short and colleagues studied *de novo* occurring genomic variants in three classes of putative regulatory elements in 7,930 individuals suffering from developmental disorders from the Deciphering Developmental Disorders (DDDs) study and their parents. The three classes of regulatory elements that they assessed consisted of 4,307 highly evolutionarily conserved non-coding elements (Siepel et al., 2005), 595 experimentally validated enhancers (Visel et al., 2007), and 1,237 putative heart enhancers (May et al., 2011), together covering 4.2 Mb of genomic sequence. In the 6,239 individuals in which exome sequencing did not find a disease cause, they found that conserved non-coding elements were nominally significantly enriched for *de novo* variants (422 observed, 388 expected, *P* = 0.04), whereas in experimentally validated enhancers (153 observed, 156 expected, *P* = 0.605), heart enhancers (86 observed, 86 expected, *P* = 0.514), and intronic controls (901 observed, 919 expected, *P* = 0.728) *de novo* variants were not enriched. When focusing only on conserved non-coding elements that had evidence of activity in brain, they observed an even stronger enrichment. Based on their analysis, the authors estimate that only around 1–3% of exome-negative individuals will be explained by *de novo* variants in fetal brain-active regulatory elements. However, as in this study only *de novo* variants were assessed, and only a limited set of regulatory elements was used which were already defined in 2010, this is likely an underestimation of the possible impact of the non-coding genome for neurodevelopmental disorders. Doan and colleagues performed a similar targeted sequencing approach assessing so-called human accelerated regions (HARs) (Doan et al., 2016). HARs are conserved regions with elevated divergence in humans and this might reflect potential roles in the evolution of human-specific traits. This study provides evidence that HARs can function as regulatory elements for dosage-sensitive genes expressed in the central nervous system. Using data from a large cohort study investigating 2100 sibling cases of ASD, they found that *de novo* CNV’s affecting HARs, or HAR-containing genes, could be implicated in up to 1.9% of ASD cases in simplex families. They then analyzed consanguineous ASD cases using WGS from 30 affected and 5 unaffected individuals and designed a custom capture array to sequence HARs in another 188 affected and 172 unaffected individuals. Individuals with ASD exhibited an excess of rare (AF < 0.5%) bi-allelic HAR alleles (43% excess compared to unaffected, *P* = 0.008), and this enrichment further increased when only taking HARs in to consideration that were likely active as regulatory elements in brain. Using MPRA, 343 bi-allelic HAR variants were functionally tested, and 29% of these were shown to alter the regulatory activity of the reference sequence. Therefore, the enrichment of regulation-altering variants in HARs with predicted activity suggests that many may contribute to the pathogenesis and diversity of ASD. They further functionally validated their findings in three examples of bi-allelic variants in HARs identified in ASD families, regulating the genes *CUX1*, *PTBP2* and *GPC4*, further providing evidence that the investigation of NCREs such as HARs is promising to solve currently genetically unexplained disease cases.

What appears from the examples given above, is that a wide range of NCRE alterations can result in effects on gene transcription, leading to a disease phenotype. This can vary from point mutations affecting the binding of crucial TFs, deletions or duplications of NCRE sequences, shuffling of the genomic location of NCREs affecting their function (e.g., enhancer adoption) or alterations in the global chromatin landscape disrupting borders of TADs, just to mention a few. Given the complexity that can exist in these disease mechanisms, and the current shortcomings in understanding the complete functionality of the non-coding genome, it seems likely that in the next decade many more examples of NCRE alteration in genetic disease will be identified.

## The Future Ahead

As we have discussed in this *Review*, our understanding of gene regulation has deepened over the last decade. NCREs have been identified as crucial modifiers of gene expression, and more and more examples of their involvement in human genetic disorders, when mutant, are being reported. In routine clinical practice genetic analysis has mainly focused on the ∼2% of the genome that directly encodes for proteins. Most of the non-coding part has been instead neglected, and only recently we could witness a shift of attention toward these sequences. It seems intuitive that, if human evolution resulted in a large and subsequently maintained expansion of the non-coding genome, this should have a functional role, and alterations of these sequences should influence their function and lead to genetic disorders. Given the fact that MCDs are often genetically unexplained despite the routine use of WES, it would be surprising if, in the near future, no genetic alterations of non-coding sequences will be identified in those currently unexplained patients. In order to achieve this, it is crucial to develop novel diagnostic approaches focusing on non-coding regions. Will WGS be useful to find disease causes in those unexplained MCD patients in a clinical setting? Theoretically yes as it will enable the identification of all detectable variants genome-wide, but our current understanding of genomic variation outside exons severely hampers its routine implementation. As a matter of fact, most studies that have used WGS in a clinical setting, have limited their analysis to those nucleotides covering exons, deep intronic variants not covered in WES and copy number and structural variants (Stavropoulos et al., 2016; Lionel et al., 2018; Clark et al., 2019; Scocchia et al., 2019). Therefore, it remains crucial to gain more detailed information on the functional relevance of NCREs and their variants from a basic science point of view. Although the characterization of epigenomic marks such as histone modifications has shown to be useful to identify functional NCREs, it is clear from the discussion above that there are still some pitfalls, as we still lack the perfect mark to identify relevant and active NCREs. One particular concern is that many studies assume that investigating a single histone modification, such as H3K27ac, gives sufficient evidence to call a region a functional NCRE, but this is certainly an oversimplification. As we have argued above, predicted NCREs should remain classified as putative NCREs till they are functionally validated, or at least predicted in multiple studies ideally using different techniques to obtain a higher level of confidence in their function. In current studies, functional validations of putative NCREs is often performed only for a selected number of regions of interest and results of these few validations are extrapolated to the complete data set generated. Even though this is understandable from a pragmatic experimental point of view, it might be one of the reasons for the broad level of variation between predicted enhancers from different sources. Hence, it is crucial to further develop high-throughput approaches for functional validation studies so that more sequences and their variants can be directly functionally tested, leading to a higher confidence in the data resources. The future application of direct functional assays, such as MPRAs and CRISPR-Cas9 based screens, is expected to further add on to our current understanding, even though also these methods are far from perfect yet. Besides these emerging experimental techniques, it is also crucial to develop novel computational tools that outperform currently available programs for NCRE prediction and disease annotation. Similarly, it is also important to further improve the linking between NCREs and their target genes, going beyond the current resolution of chromatin conformation capture or correlation between activity of putative enhancers and expression of possibly linked genes. Until we will have all these ideal tools widely available, in our opinion the best practice to study the role of the non-coding genome in genetic disorders such as MCDs is to study genetic variation outside exomes in well-defined, exome negative patients and preferably combine this with a direct readout of gene expression in a disease relevant tissue. For the functional annotation of the non-coding variants found in patients, it is essential to use as many sources of information as possible, enabling the highest level of confidence in defining a certain region a regulatory sequence. And last but certainly not least, a detailed clinical phenotyping of patients prior to any genetic investigation remains crucial as it allows the comparison of patients with similar non-coding variants and shared phenotypes. Even in an era where it is cheap to sequence a whole genome, reverse phenotyping of patients remains essential to learn more about the consequences of the genetic variants and to further mature our understanding of the non-coding genome beyond the borders of the exome.

## Author Contributions

EP, SY, and EN performed the literature research and wrote the sections of the manuscript. TB conceived the manuscript, wrote the sections, supervised the work, and obtained funding. All authors approved the final version of the manuscript.

## Conflict of Interest Statement

The authors declare that the research was conducted in the absence of any commercial or financial relationships that could be construed as a potential conflict of interest.
